# Association between caregivers’ knowledge and care seeking behaviour for children with symptoms of pneumonia in six sub-Saharan African Countries

**DOI:** 10.1186/s12913-017-2060-3

**Published:** 2017-02-02

**Authors:** Aaltje Camielle Noordam, Alyssa B. Sharkey, Paddy Hinssen, GeertJan Dinant, Jochen W. L. Cals

**Affiliations:** 1 CAPHRI School for Public Health and Primary Care, Maastricht University, P.O. box 616, Maastricht, The Netherlands; 2 United Nations Children Fund (UNICEF), Three United Nations Plaza, New York, NY 10017 USA

## Abstract

**Background:**

Pneumonia is the main cause of child mortality world-wide and most of these deaths occur in sub-Saharan Africa (SSA). Treatment with effective antibiotics is crucial to prevent these deaths; nevertheless only 2 out of 5 children with symptoms of pneumonia are taken to an appropriate care provider in SSA. While various factors associated with care seeking have been identified, the relationship between caregivers’ knowledge of pneumonia symptoms and actual care seeking for their child with symptoms of pneumonia is not well researched.

**Methods:**

Based on data from Multiple Indicator Cluster Surveys, we assessed the association between caregivers’ knowledge of symptoms related to pneumonia – namely fast or difficulty breathing – and care seeking behaviour for these symptoms. We analysed data of 4,163 children with symptoms of pneumonia and their caregivers. A Chi-square tests and multivariable logistic regression was performed to assess the association between care seeking and knowledge of at least one symptom (i.e., fast or difficulty breathing).

**Results:**

Across all 6 countries only around 30% of caregivers were aware of at least one of the two symptoms of pneumonia (i.e., fast or difficulty breathing). Our study shows that in the Democratic Republic of the Congo and Nigeria there was a positive association between knowledge and care seeking (*P* ≤ 0.01), even after adjusting for key variables (including wealth, residence, education). We found no association between caregivers’ knowledge of pneumonia symptoms and actual care seeking for their child with symptoms of pneumonia in Central African Republic, Chad, Malawi, and Sierra Leone.

**Conclusions:**

These findings reveal an urgent need to increase community awareness of pneumonia symptoms, while simultaneously designing context specific strategies to address the fundamental challenges associated with timely care seeking.

## Background

Pneumonia is responsible for more deaths among children under five years of age than any other infectious disease. In 2015, pneumonia killed an estimated 922,000 children under-five globally; most of these deaths were in sub-Saharan Africa [1]. Timely treatment with effective antibiotics is critical to prevent pneumonia-related deaths [2].

Unfortunately, early care seeking for childhood illnesses remains a challenge in countries with high mortality rates [3–7]. Estimates from sub-Saharan Africa indicate that only 2 out of the 5 children with pneumonia specific symptoms are taken to an appropriate provider for care [1]. Overall, poorer and less educated households are less likely to seek care [3, 6, 7]. Other studies conducted across sub-Saharan Africa also identified cultural beliefs, religion, habit, perceived severity of the illness, and previous experiences with health services as key factors which influence the decision of caregivers to seek care [3, 7–9]. Besides these factors, the role and ability of the primary caregiver to decide to seek care, the associated cost, as well as the distance to the health services influences the timeliness in which care is received [3]. In some countries the age and the sex of the child can also influence care seeking; a study found that in Nigeria, Ethiopia and the Democratic Republic of the Congo (DRC) children less than 24 months were more likely to be brought for care than those between 24 and 60 months, and in Uganda girls were more likely to be brought for care as supposed to boys [7].

Moreover as failure to recognize an illness is expected to lead to delays in care seeking, the first step to address pneumonia specific mortality is by ensuring that caregivers are aware of pneumonia specific symptoms [10]. To date, the relationship between specific knowledge of these symptoms, recognition of them and related care seeking is not well researched [8]. And, only a few studies specifically focus on the association between knowledge of symptoms related to pneumonia – namely fast or difficulty breathing – and care seeking behaviour [11, 12]. We hypothesized that knowledge of these specific symptoms will enable caregivers to recognize an illness, consequently seeking timely and appropriate care. Such information is critical to plan effective strategies to reduce pneumonia mortality in high-burden settings, particularly where rates of care seeking are inadequate.

## Methods

### Data sources

Our analyses were based on data from Multiple Indicator Cluster Surveys (MICS) conducted in sub-Saharan Africa. These nationally representative household surveys are conducted by national implementing agencies with the support of the United Nations Children’s Fund (UNICEF). MICS surveys collect statistically sound and internationally comparable data on a variety of topics related to maternal and child health, including knowledge of symptoms of childhood illnesses and care seeking behaviour for children under the age of five years [13, 14]. Sub-Saharan African countries were selected for inclusion in this study if they had a MICS conducted during or after 2010, the data was national representative, and the datasets were available upon the start of our analyses (August 2015). Information on knowledge of symptoms of childhood illnesses was obtained during interviews using the ‘*individual women’s*’ questionnaire, administered to women age 15 through 49 years. Data on care seeking behaviour were obtained via the ‘*children under-five*’ questionnaires, administered primarily to mothers of children under the age of five years. When the mother was deceased or living elsewhere, the questionnaire was administered to the child’s primary caregiver.

### Survey methods

Sampling frames are usually based on the most recent national census and therefore do not include non-household populations; i.e., they exclude populations living in group quarters (e.g., hospitals, military barracks) and those living on the street. Usually, a two-stage cluster sampling approach is used; the first stage: select enumeration areas and do a listing of households, second stage: select households from list. While the surveys we used were conducted in different countries - and may therefore vary due to limitations in costs and practical considerations (including security) - all surveys ‘*adhere to the fundamentals of scientific sampling, including complete coverage of the targeted population, use of suitable sample size, the need to conduct household listing and pre-selection of sample households*’ [13].

### Research questions

For each country, we first assessed the proportion of caregivers (either mothers, or primary caregivers) of children under-five that mentioned one or both symptoms of childhood illnesses linked to pneumonia, i.e., fast and/ or difficulty breathing, as a reason to seek care. The specific interview question asked to caregivers was: “*Sometimes children have severe illnesses and should be taken immediately to a health facility. What types of symptoms would cause you to take a child under the age of 5 to a health facility right away?”* A subsequent probe question asked: *“Any other symptoms?*” The responses were categorized as: child is not able to drink or breastfeed; becomes sicker; develops a fever; has fast breathing; has difficulty in breathing; has blood in stools; is drinking poorly; any other (unspecified); and some countries included categories such as diarrhoea and/ or vomiting. We classified caregivers who were able to identify either fast or difficulty breathing as having knowledge of pneumonia symptoms. As responses were not categorized as “cough” or “chest in-drawing,” we were not able to include these symptoms in our analysis of pneumonia specific knowledge.

Second, we calculated how many of these caregivers reported that their child had a cough and fast or difficulty breathing due to a problem in the chest in the past two weeks, as cases with symptoms of pneumonia. The interview questions related to these cases are: “*Has (name) had an illness with cough at any time in the last 2 weeks?” “When (name) had an illness with cough, did he/she breathe faster than usual with short rapid breaths or have difficulty breathing?” “Was the fast or difficult breathing due to a problem in the chest or to a blocked or runny nose?*” The children of whom the caregiver reported that they had a cough with fast or difficulty in breathing, due to a problem in the chest in the past two weeks, where considered as cases with symptoms of pneumonia.

Third, we assessed which proportion of these caregivers brought their child to an appropriate health provider. The interview questions related to care seeking were: “*Did you seek advice or treatment for the illness from any source?” “Where did you seek advice or treatment?”* A subsequent probe asked, “*Anywhere else*?” We defined ‘appropriate’ health care provider as one working at either a private or public hospital, primary health care facility or any other government service, and who has undergone formal training and received accreditation, authorizing them to treat children with signs of acute respiratory tract infections. Once we knew which proportion sought appropriate care, we assessed if the caregivers who mentioned at least one pneumonia specific symptom as reason to seek immediate care – i.e., fast or difficulty breathing – were indeed more likely to seek care from these providers, as supposed to those who did not mention one of these symptoms.

### Data analysis

We conducted our analyses using SPSS version 21. The data analysis was conducted by two independent researchers [CN and PH]. We first created a new dataset by merging the two (i.e., the ‘*individual women’s*’ and ‘*children under-five*’) data files, matching eligible cases for both surveys based on cluster, household and line number. For caregivers with more than one child under the age of five, we used the files of the youngest child - in the case of twins we kept the child that was mentioned last. We weighted the data using the sample weight variables. Cases with missing data (i.e., surveys which were partly completed, as well as missing variables needed for these analyses) were excluded from the analyses. We calculated the percentage of caregivers included in the merged dataset who reported fast or difficulty breathing as reasons to seek immediate care from a health facility. We then calculated cross tabulations and performed Chi-square tests to assess the association between care seeking and knowledge of at least one symptom (i.e., fast or difficulty breathing).

Based on the literature on care seeking, we included the following variables in the multivariate analyses: household wealth quintile (poorest, poorer, middle, richer and richest); residence (rural, urban); caregiver’s age (15–19, 20–24, etc.) and their level of education (none, primary, and secondary or higher); child age (<2 years, 2–5 years) and their sex (boy, girl); and the total number of children ever born (<2, 2–3 and 4+). When possible we also adjusted for geographical location (regions) and religion. With the categories for the last two variables being country specific, not always available, and – in some cases – sample size restrictions, we pre-defined some parameters: 1) the sample had to be large enough (≥10 cases per category); 2) geographical location had to refer to regions e.g., the North, East, South or West and not to any other groupings (i.e., provinces, or districts), and 3) we first assessed if we could include geographical location (regions), after which we conducted the same assessment for religion.

We performed a multivariate logistic regression for the dependent variable (care seeking from an ‘appropriate’ health provider) and the independent variables, in order to examine the association. We then calculated the adjusted odds ratios (ORs) with corresponding 95% confidence intervals (CIs).

## Results

Of countries in sub-Saharan Africa, in which a MICS was conducted during or after 2010 and for which survey results were available before the start of our analyses, we selected the six countries with the largest sample sizes for analysis: Central African Republic (CAR), Chad, Democratic Republic of the Congo (DRC), Malawi, Nigeria, and Sierra Leone. Four surveys were from 2010 (CAR, Chad, DRC and Sierra Leone), Nigeria’s survey was from 2011 and Malawi’s was from 2013–14.

### Background characteristics

Table 1 shows the samples per country, after merging the women’s and children’s datasets, with Nigeria as the largest sample (*N* = 16,242) and Sierra Leone as the smallest (*N* = 6,033). The vast majority of caregiver-child combinations included in the analyses across the six countries live in rural settings, and most of the caregivers have at least four children. The largest differences across the countries were found in levels of education, with most caregivers having had no education in Chad (73.8%), Sierra Leone (71.2%) and Nigeria (42.0%) and at least primary school in Malawi (69.6%), DRC (42.7%) and CAR (42.6%). The percentage of cases with symptoms of pneumonia (i.e., those for whom the mother or caregiver mentioned that their child had a cough and fast and/or difficulty breathing due to a problem in the chest in the past two weeks) ranged from 3.7% (*n* = 607) in Nigeria to 9.6% (*n* = 947) in Chad (Table 1). Hence, in the total population we had 4,163 cases with symptoms of pneumonia.

**Table 1:** Background characteristics of children

	CAR		Chad		DRC		Malawi		Nigeria		Sierra Leone	
	%	n	%	n	%	n	%	n	%	n	%	n
Wealth index												
Poorest	21.6	1475	19.2	1897	22.5	1600	22.1	3079	22.8	3697	22.4	1349
Poor	21.7	1478	20.2	1992	20.8	1484	21.6	3014	20.8	3377	21.4	1292
Middle	20.4	1389	21.0	2078	20.5	1459	20.4	2851	18.6	3028	20.6	1245
Rich	19.5	1328	21.5	2123	19.6	1394	18.1	2522	18.9	3071	19.6	1185
Richest	16.9	1151	18.1	1784	16.6	1185	17.8	2486	18.9	3070	16.0	963
Residence												
Rural	64.1	4371	78.5	7755	73.5	5238	87.0	12137	68.9	11188	72.1	4349
Urban	35.9	2450	21.5	2118	26.5	1884	13.0	1814	31.1	5054	27.9	1684
Education level												
None	41.4	2823	73.8	7289	24.1	1715	12.0	1670	42.0	6828	71.2	4295
Primary	42.6	2905	18.4	1821	42.7	3044	69.6	9702	19.7	3202	13.5	813
Secondary +	16.0	1093	7.7	763	33.2	2363	18.4	2574	38.2	6212	15.3	925
Number of children												
0-1	18.5	1261	14.2	1403	16.9	1205	19.7	2754	15.7	2551	18.5	1119
2-3	34.9	2378	27.6	2728	30.5	2173	35.5	4947	31.9	5189	34.6	2090
4+	46.6	3182	58.2	5742	52.6	3744	44.8	6250	52.3	8502	46.8	2824
Child age												
< 2 years	62.2	4241	58.9	5820	65.8	4687	50.3	7015	60.7	9858	50.7	3058
2-5 years	37.8	2580	41.1	4053	34.2	2436	49.7	6936	39.3	6384	49.3	2975
Sex of the child												
Male	50.6	3449	49.8	4920	50.2	3575	50.6	7058	51.4	8341	50.3	3031
Female	49.4	3372	50.2	4954	49.8	3548	49.4	6892	48.6	7901	49.7	2998
Total sample size		6821		9873		7123		13951		16242		6033
Total reported cases^a^	7.2	492	9.6	947	6.6	473	7.8	1088	3.7	607	9.2	556

*Note*: For all calculations, numbers and percentages are based on weighted averages which are also adjusted for missing data. For these reasons, manual re-calculation might show slight differences (i.e., the total sample size will vary for each category as the missings within this category will differ). ^a^Cases are children with symptoms of pneumonia in the past two weeks, as reported by their caregiver

### Knowledge of fast and/or difficulty in breathing

Of the two symptoms linked to pneumonia, caregivers were most aware of the symptom ‘difficulty in breathing’, ranging from 17.4% of the sample in Chad to 24.0% in CAR, see Table 2. Across all 6 countries around 30% (ranging from 29.2 - 32.9%) of caregivers were aware of at least one of the two symptoms. We characterised these caregivers as caregivers with appropriate knowledge of pneumonia symptoms. When assessing the percentage of caregivers who mentioned both symptoms, this ranged from 4.5% in Chad to 11.2% in CAR.

**Table 2:** Percentage of caregivers surveyed who had knowledge of pneumonia symptoms

	CAR	Chad	DRC	Malawi	Nigeria	Sierra Leone
Symptoms mentioned as reason to seek care						
Difficulty breathing	24.0%	17.4%	20.6%	19.9%	22.7%	21.7%
Fast breathing	17.0%	16.3%	16.0%	14.8%	19.5%	19.5%
Fast *and* difficulty in breathing	11.2%	4.5%	6.8%	5.1%	10.3%	8.3%
Fast *or* difficulty in breathing (defined as “knowledge” in this paper)	29.7%	29.2%	29.7%	29.6%	31.9%	32.9%
Total number of survey sample	6821	9873	7123	13951	16242	6033

### Care seeking behaviour

Table 3 shows the associations between knowledge of at least one symptom for pneumonia and care seeking from appropriate health care providers, also with adjustments for the predefined variables in a multivariate logistic regression model. Of children with symptoms of pneumonia in the previous two weeks, those living in Sierra Leone and Malawi were most likely (73.2% and 68.8%, respectively) to be brought to an appropriate provider. In Chad and CAR this was only 27.4% and 30.9%, respectively. Care seeking was only moderately better in Nigeria (41.9%) and DRC (44.2%).

**Table 3:** Associations between knowledge of at least one symptom for pneumonia and care seeking from appropriate health care providers

	Children with symptoms of pneumonia, taken to an appropriate provider			
	Total taken to provider and reported knowledge of pneumonia symptom	N (%)	Unadjusted OR + (95% CI)	Adjusted^a^ OR + (95% CI)
CAR	Total	151/ 489 (30.9)		
	Knowledge - No	104/ 357 (29.1)	ref	ref
	Knowledge - Yes	47/ 132 (35.6)	1.3 (0.9-2.1)	1.4 (0.9 – 2.2)
Chad	Total	254/ 928 (27.4)		
	Knowledge - No	166/ 630 (26.3)	ref	ref
	Knowledge - Yes	88/ 298 (29.5)	1.2 (0.9 – 1.6)	1.1 (0.8 – 1.6)
DRC	Total	209/ 473 (44.2)		
	Knowledge - No	120/ 309 (38.8)	ref	ref
	Knowledge – Yes	89/ 164 (54.3)	1.9 (1.3 – 2.7)**	2.0 (1.3 – 3.0)**
Malawi	Total	740/ 1076 (68.8)		
	Knowledge - No	519/ 743 (69.9)	ref	ref
	Knowledge - Yes	221/ 333 (66.4)	0.9 (0.6 – 1.1)	0.8 (0.6 – 1.1)
Nigeria	Total	253/ 604 (41.9)		
	Knowledge - No	155/ 405 (38.3)	ref	ref
	Knowledge - Yes	98/ 199 (49.2)	1.6 (1.1-2.2)**	1.9 (1.3 – 2.9)**
Sierra Leone	Total	402/ 549 (73.2)		
	Knowledge - No	267/ 369 (72.4)	ref	ref
	Knowledge - Yes	135/ 180 (75.0)	1.1 (0.8 – 1.7)	1.2 (0.8 – 1.9)

*Note*: Knowledge is defined as those caregivers aware of at least one pneumonia symptom (i.e., fast *or* difficulty breathing). All calculations: numbers (*n*), percentages (%), odds ratios (OR) and 95% confidence intervals (CI) are based on weighted averages, adjusted for missing data. Numbers presented in this table are rounded. Manual re-calculation might therefore show slight differences

^a^Adjusted for pre-defined variables, namely; wealth, residence, maternal education, maternal age, child’s age, child’s sex, and total number of children ever born. Based on the pre-defined criteria geographical location (defined as regions) were included in Malawi, Nigeria and Sierra Leone and religion in Chad, Malawi, and Nigeria

Statistical significance: *p ≤ 0.05, **p ≤ 0.01

In DRC, the odds of a child whose caregiver mentioned at least one symptom related to pneumonia as reasons to seek immediate care was 1.9 times higher (95% CI = 1.3-2.7, *P* ≤ 0.01) than the odds for a child whose caregiver did not mention one of the symptoms to have been brought to an ‘appropriate’ health provider during his or her last illness. The association remained largely the same after adjusting for wealth, residence, maternal age and education, the age and sex of the child, and number of children ever born (OR = 2.0, 95% CI = 1.3-3.0, *P* ≤ 0.01). A significant association was also found in Nigeria, where the odds of a caregiver with knowledge of symptoms was 1.6 times higher (95% CI = 1.1-2.2, *p* ≤ 0.01) as opposed to the odds of a caregiver without knowledge of symptoms to seek care from an ‘appropriate’ health provider. After adjusting for the pre-defined variables as mentioned for DRC, and for religion (Muslim, Christian) and geographical location (6 regions; north-east, north-west, south-south, south-east, south-west, north-centre) the OR increased to 1.9 (95% CI = 1.3-2.9, *p* ≤ 0.01). For the other four countries, the analyses revealed no significant association between knowledge and care seeking.

## Discussion

In this study, we found low levels of caregivers’ knowledge of symptoms related to childhood pneumonia in sub-Saharan Africa; on average only around 30% of caregivers were aware of one of the pneumonia symptoms, namely either fast or difficulty in breathing. However, these caregivers with knowledge of pneumonia symptoms were not necessarily more likely to seek care from an appropriate health care provider. We found a significant, positive association between knowledge of at least one symptom and care seeking in DRC and Nigeria. This association was evident even after adjusting for key background characteristics. For the other four countries (CAR, Chad, Malawi and Sierra Leone) the associations were not significant.

Low levels of knowledge of pneumonia specific symptoms are confirmed by other studies, even compared to knowledge of other illnesses such as malaria and diarrhoea [15, 16]. Of the two symptoms included in this study, we found that caregivers more frequently mentioned difficulty in breathing as compared to fast breathing. A study conducted in Nigeria found that mothers were more likely to recognise pneumonia based on fever and cough, than on either fast or difficulty breathing [17]. This same study also found that mothers were less likely to recognize pneumonia based on more severe symptoms such as chest in-drawing and central cyanosis [17]. Studies conducted in Sierra Leone [18] and Nigeria [11] also report a lack of knowledge on symptoms and that caregivers are often not aware of ways to prevent or treat common childhood illnesses, including pneumonia.

Despite the importance of increasing knowledge on pneumonia and its symptoms, we only found a significant, positive association between knowledge of pneumonia symptoms and care seeking in two out of the six countries. These findings underscore the main findings of other studies which highlighted the importance of understanding country-specific care seeking patterns and the related determinants, especially with care seeking being a dynamic process influenced by a range of socio-economic, cultural and geographic access factors [3–7, 19–21]. More specifically, a study from Malawi reported that wealth, urban–rural residence, and maternal education were associated with care seeking [22], illustrating that to overcome these challenges knowledge on pneumonia alone was insufficient.

For the above reasons, it is too early to say which impact knowledge of pneumonia symptoms has on timely care seeking. With studies reporting that care seeking is linked to caregivers’ perception of the severity of the illness: the more severe caregivers perceived the illness, the more likely they were to seek care [8, 23]. Further research needs to examine how knowledge of pneumonia symptoms affects early recognition of the primary symptoms of pneumonia. Furthermore, with knowledge of pneumonia symptoms being low across these six countries (ranging from 29.2 to 32.9%), a similar study across countries with higher levels of knowledge on pneumonia and care seeking could help get a better understanding of the added value of large campaigns aiming to increase knowledge of the main cause of childhood mortality worldwide. Finally, to help better understand country-specific challenges associated with care seeking, questions on why care is (or is not) sought should be included in national household surveys; such data is crucial to help prioritize health strategies.

While the low level of association between knowledge and care seeking is concerning, other studies have shown that knowledge alone is insufficient to change behaviour; e.g., knowledge of the importance of using a condom to reduce the risk of HIV transmission, does not necessarily translate to higher use of condoms amongst those most at risk [24]. Other studies also found that community based approaches such as provision of integrated case management of childhood illnesses - including pneumonia - can be an important strategy [25, 26]. A study from Nigeria [27] showed that community information activities for malaria lead to improved knowledge, home management and referral practices.

### Limitations

The main limitations of this study are linked to the fact that the reported prevalence, knowledge and care seeking patterns are based on questions to the primary caregiver. For example, the children with symptoms of pneumonia are based on caregivers’ perceptions of symptoms and their ability to recall events, which could lead to incorrect estimates [28]. In other words, if caregivers do not recognize the symptoms of children which really have pneumonia, the cases are not categorized as ‘children with symptoms of pneumonia’. In relation to this, we were not able to assess if knowledge of pneumonia specific symptoms lead to more accuracy in the recognition of pneumonia specific symptoms, subsequently leading to better identification of suspected cases, or, as the cases are not clinically validated as those requiring medical care, not seeking care might in some cases be justified. Also, we were not able to disaggregate the data by severity of the illness, or by the severity of symptoms (e.g., chest in-drawing), or if a child had multiple symptoms; all factors which are known to be associated with care seeking. Plus, as caregivers were not prompted about specific symptoms, it is possible that pneumonia specific symptoms just did not come to mind when interviewed, this could have led to under-reporting of these specific symptoms. Finally, we were not able to discern where caregivers learned about symptoms of childhood illnesses and whether or not they learned this after care seeking for the child’s last illness. A study from Nigeria [29] showed that only 23% of the caregivers received health information from the healthcare providers after being hospitalized.

## Conclusion

We found low levels of knowledge on pneumonia symptoms among caregivers in six high burden countries in sub-Saharan Africa, as well as limited positive associations between knowledge and care seeking. Therefore, despite the fact that we need to increase knowledge of pneumonia symptoms, our study shows that this alone is not sufficient to take action. With low levels of knowledge of symptoms of pneumonia across these high pneumonia mortality settings, emphasis should be put on education programs, which not only focus on the primary caregivers, but on all those involved in decision-making processes and care seeking. In addition, factors other than knowledge (e.g., empowerment, costs) should also be addressed to improve care seeking behaviour. In other words, there is a need to increase community awareness of pneumonia, while simultaneously designing context specific strategies to address the fundamental challenges associated with timely care seeking.

## Abbreviations

CAR: Central African Republic
CIs: Confidence Intervals
DRC: Democratic Republic of the Congo
MICS: Multiple Indicator Cluster Surveys
ORs: Odds ratios
SSA: Sub-Saharan Africa
UNICEF: United Nations Children’s Fund
